# Artificial Intelligence and Machine Learning for Automated Cephalometric Landmark Identification: A Meta-Analysis Previewed by a Systematic Review

**DOI:** 10.7759/cureus.40934

**Published:** 2023-06-25

**Authors:** Sabita Rauniyar, Sanghamitra Jena, Nivedita Sahoo, Pritam Mohanty, Bhagabati P Dash

**Affiliations:** 1 Orthodontics and Dentofacial Orthopaedics, Kalinga Institute of Dental Science, Bhubaneswar, IND; 2 Department of Orthodontics and Dentofacial Orthopaedics, Kalinga Institute of Dental Sciences, Kalinga Institute of Industrial Technology (KIIT) (Deemed to be University), Bhubaneswar, IND; 3 Department of Orthodontics, Kalinga Institute of Dental Sciences, Odisha, IND

**Keywords:** manual tracing, cellular neural network, machine learning, automated cephalometric landmark, : artificial intelligence

## Abstract

Digital dentistry has become an integral part of our practice today, with artificial intelligence (AI) playing the predominant role. The present systematic review was intended to detect the accuracy of landmarks identified cephalometrically using machine learning and artificial intelligence and compare the same with the manual tracing (MT) group. According to the PRISMA-DTA guidelines, a scoping evaluation of the articles was performed. Electronic databases like Doaj, PubMed, Scopus, Google Scholar, and Embase from January 2001 to November 2022 were searched. Inclusion and exclusion criteria were applied, and 13 articles were studied in detail. Six full-text articles were further excluded (three articles did not provide a comparison between manual tracing and AI for cephalometric landmark detection, and three full-text articles were systematic reviews and meta-analyses). Finally, seven articles were found appropriate to be included in this review. The outcome of this systematic review has led to the conclusion that AI, when employed for cephalometric landmark detection, has shown extremely positive and promising results as compared to manual tracing.

## Introduction and background

Dentistry as a science has evolved by leaps and bounds, and particularly orthodontics as a specialty has evolved spectacularly. Integration of orthodontics, AI, and ML has shown promising results [1]. The use of technology in the form of AI to simulate a homo sapiens brain to execute functions using a computer-generated algorithm is a relatively new concept with far-fetched results [2,3]. Although AI development had begun in 1943, the term AI was formally declared in 1956 at a conference at Dartmouth by John McCarthy [4,5]. AI has enormous potential not only in predicting, organizing, or representing information but also in creating a sustainable treatment plan using an algorithm to benefit practitioners not only in the medical fraternity but also has spread its tentacles into every branch of science [6,7]. Both deep learning and machine learning are subsets of artificial intelligence that use algorithms to create neural networks that perform like a human brain to the best of their ability, thus duplicating human presence and eliminating manual errors [8-10].

The present scenario demands the essential use of AI in the ever-evolving branch of dentistry, not only as an important diagnostic aid but also as a powerful decision-making tool [11]. AI has manifold uses, beginning from diagnosis to detection of cephalometric landmarks [5], classification of maxillofacial cysts and tumors [12,13], and the description of periapical diseases [14]. AI provides a solution for every problem [15].

**Figure 1:**
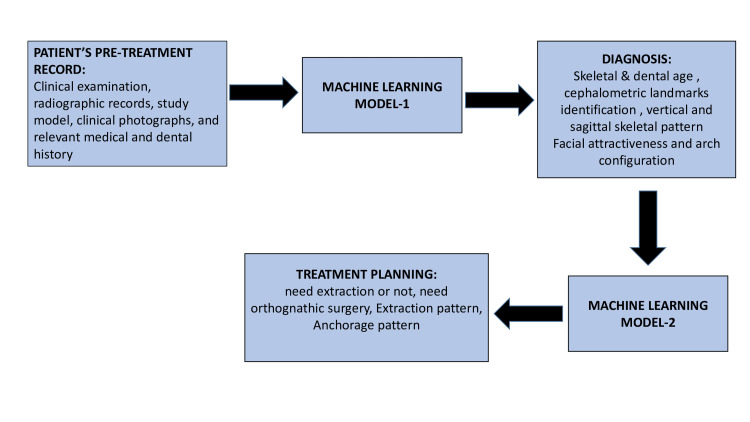
Graphical representation of machine learning.

In order to evaluate problems related to orthodontics, AI provides the most efficient and cost-effective solutions. In order to treat orthodontic patients, whether to follow the extraction protocol or the non-extraction protocol depends not only on clinical experience but also on evidence based on accurate diagnostic aids [16,17]. Thus, we can say that deep-learning methods can provide an effective solution to the problem [18]. When the treatment of a patient is planned for combined orthodontic and surgical therapy, AI provides ample potential [19,20]. Furthermore, in order to detect anomalies in facial growth [21], cephalometry is widely employed, but identifying the landmarks using the manual method not only consumes time but also includes a high risk of errors [22]. Many studies done during the past few years using AI for automated landmark detection have demonstrated outstanding achievements [5,8]. Moreover, if one wants to predict facial aesthetics following orthodontic therapy in combination with orthognathic surgery, using AI will be an optimal choice prior to the surgical procedure. Figure 1 is a graphical representation of the use of AI and ML in orthodontics [6].

The present systematic review aimed to detect the accuracy of landmarks identified with cephalometry using artificial intelligence and machine learning and compare the same with the manual tracing (MT) group.

## Review

​​​​Priority Reporting Items of the Extended Meta-Analysis for Systematic Reviews and Diagnostic Test Accuracy (PRISMA-DTA) guidelines was performed as per the protocol for any systematic review and is registered in the National Institutes of Health database (www.crd). york.ac.uk/Prospero, logs: CRD42022380990)

"PICO" format for the conduction of study: Participants: Patient who came for orthodontic treatment. Intervention: lateral cephalograms. Comparison: tracing between AI and manual. Outcomes: to predict accuracy.

The following measures were undertaken for the review: Inclusion Criteria were examination of diagnostic accuracy using AI and machine learning, train and test using 2D cephalometric images, a minimum no. of 5 landmarks that are relevant for the study to be recognized and published articles in English from 2001 to 2022. Studies with non-radiological data, cephalometrically irrelevant landmarks, or lack of AI or machine learning techniques were excluded. 

Guidelines for search strategy and study selection: A period ranging from January 2001- November 2022) spanning over a period of twenty years were searched digitally. The various search engines employed for the study included PubMed, Embase, Google Scholar, Scopus, and Doaj published over the past two decades. We restricted ourselves to articles printed using the British English language for publication. Unpublished literature was searched electronically by keywords such as artificial intelligence and cephalometric landmarks, machine learning and cephalometric landmarks, and ‘automatic detection of cephalometric landmarks and orthodontics’. 

Data extraction was recorded according to the 'PICO' guidelines. Information collected included the author’s name, the year in which it was published, the country where the study was conducted, whether it was published in a specialty journal of orthodontics, and the target number of people involved in the study, type of procedure, comparative goals, the outcome of the procedure, and the kind of deep learning (AI algorithms) used. 

Results

Full-length abstracts and papers were retrieved. The data required for the review were selected in two steps. During the first round, only those articles whose titles and abstracts were tallied with the review topic were selected. The outcome of this resulted in 137 papers on which we carried out the study. Eighty papers were removed as irrelevant to the study title, and twenty duplicate records were excluded. A total of 37 items were included in the next round of reviews. Once the above-selected articles were reviewed, irrelevant and similar articles were excluded based on the set exclusion and inclusion criteria. The number of articles was further reduced to 13 after applying these criteria. Author names and journal names were masked and circulated among authors. Six full-text articles were also excluded (three articles did not provide a comparison of manual tracking and AI on cephalometric landmark detection, and three full-text articles were systematic reviews and meta-analyses). Finally, this systematic review included seven papers on qualitative synthesis. Each of these papers has been extensively researched and quantified by year of publication to develop trends that have emerged in the field of artificial intelligence in dentistry in recent years.

**Figure 2:**
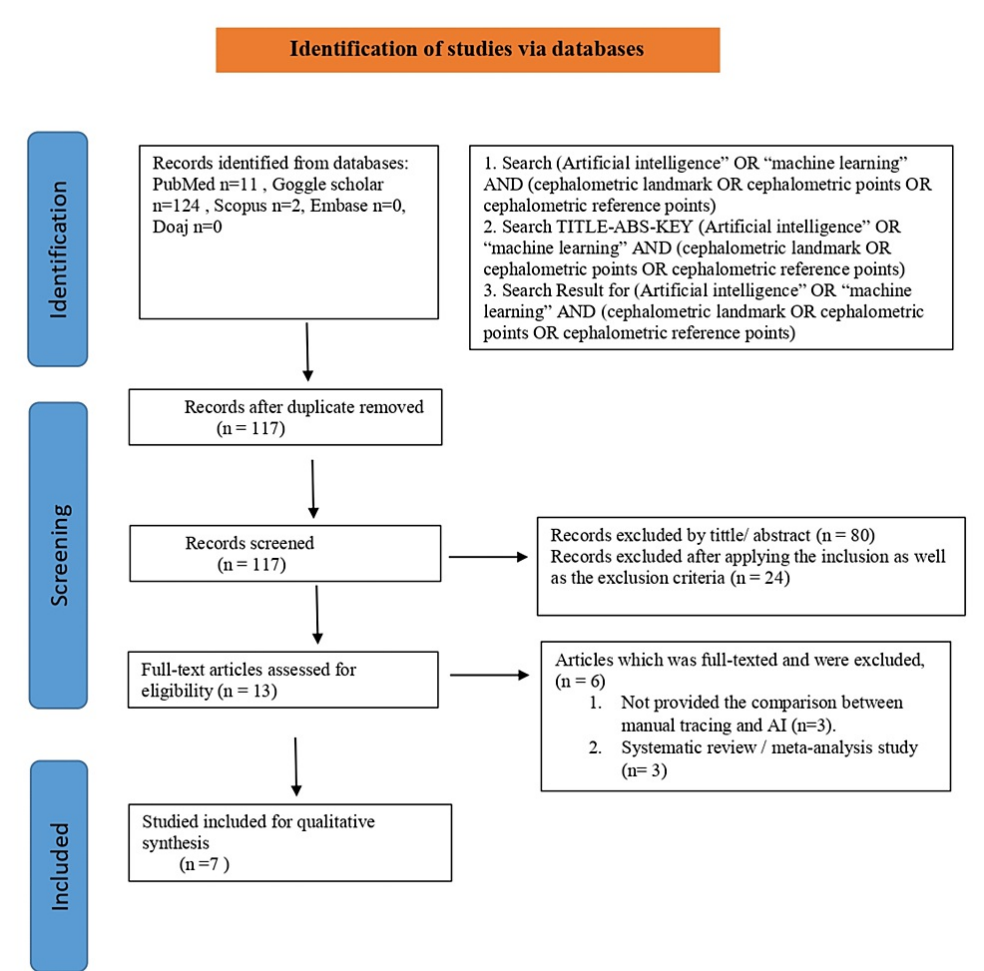
Screening and selection of articles flowchart.

The search resulted in a total of 137 articles. Of these, thirty-seven articles were checked for full text, and 13 were ultimately included in this review. Out of 13 articles, six full-text articles were excluded. A flowchart details the reasons for including and excluding studies (Figure 2). The study was conducted in Spain, Taiwan, South Korea, and the United States and was published over a 21-year period (i.e., 2001-2022). A brief description of the study characteristics is summarized in Table 1.

**Table 1:** Characteristics of study articles.

1st author	Year	Country	Imagery	Data source	No. landmarks	Total sample	Performance measurements	
							2-mm SDR	MRE (mm)
Grau et al. [23]	2001	Spain	Radiographs	Unclear	17	20	90.30%	-
Lindner et al. [24]	2016	Taiwan	Lateral Cephalograms	Unclear	19	400	84.70%	-
Hwang et al. [5]	2020	South Korea	X-ray images	Unclear	80	1311	-	
Kim et al. [25]	2021	South Korea	Radiographic images	Yonsei University Dental Hospital dataset	13	950	64.30%	1.84
Bulatova et al. [26]	2021	USA	X-ray images	Website from AAOF Legacy Denver	16	110	80.4	-
Tsolakis et al. [4]	2022	Unclear	CS8100SC Evo Edition X-rays	Own data	18	100	-	-
Le et al. [27]	2022	South Korea	Lateral cephalograms	Korean healthcare company in Seoul at JNU Dental Hospital. Children between 6 and 18 years from the OPD of the pedodontics department between 2008 and 2018.	41	1293	73.32%	1.87

Risk of Bias

**Figure 3:**
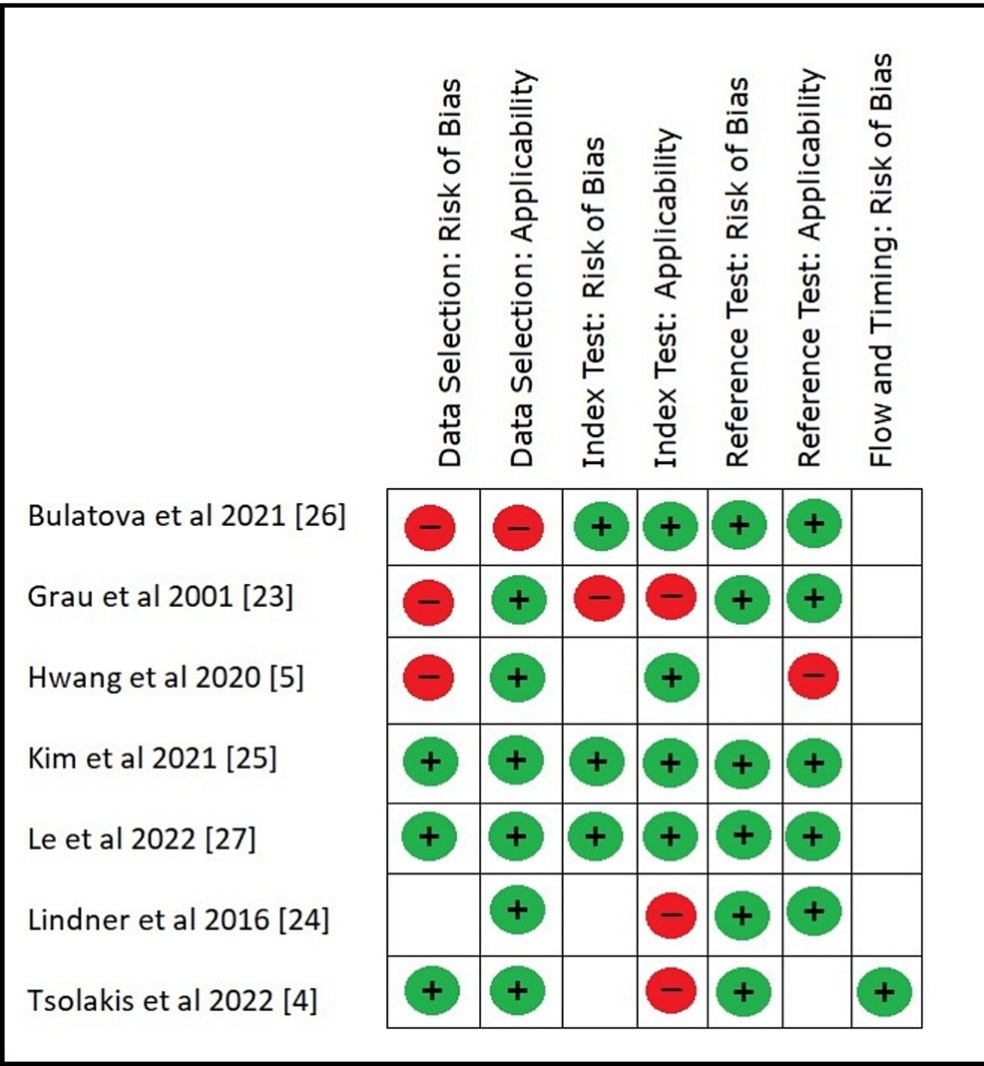
Evaluation of the risk of prejudice and the degree of appropriateness.

In this study, four main domains - (1) selection of data, (2) index test, (3) reference test, and (4) process and time consumed - were evaluated for the risk of bias. Low values ​​were found in most studies. Regarding applicability, most (four) studies showed high risk. More information on the evaluation of the risk of prejudice and the degree of appropriateness is shown in Figure 3.

Meta-Analysis

Among the seven eligible articles, five of them could be taken into the meta-analysis, and the rest were excluded due to the absence of data homogeneity. Based on the successful detection rate (SDR), a meta-analysis was conducted, which helps assess the efficiency of artificial intelligence (AI). SDR is a ratio of the variation between predicted and reference landmarks within a specified distance. When this difference was within 2 mm, it was accepted as a successful detection by the AI. Though the SDR was calculated for different discrepancies, such as 2 mm or 2.5 mm or 3 mm, or even 4 mm, in different articles, we used the data for the SDR (at the 2 mm prediction error threshold) only for this meta-analysis, which was presented as a percentage in all five articles.

The included studies expressed the SDR in percentage, and the number of included samples was described in their methods. From these two numbers, we calculated the number of successful cases where the AI predicted the values correctly by remaining within the permissible margin of error (2.0 mm).

**Figure 4:**
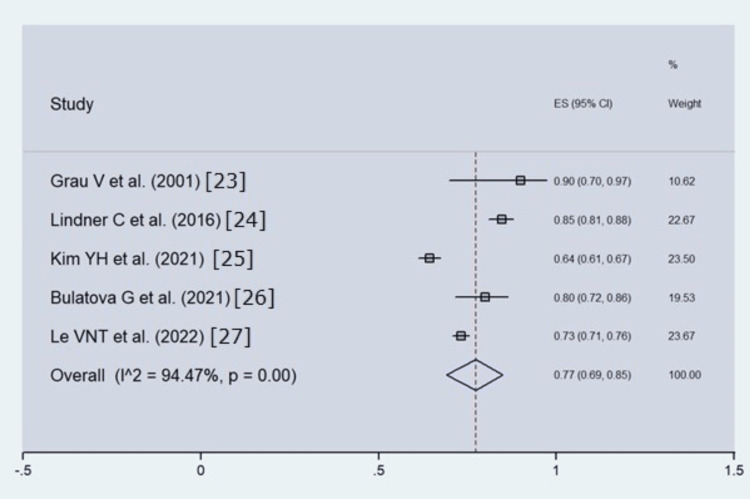
Forest plot of showing SDR from a prediction error threshold of 2 mm. Squares indicate the SDR for each individual study and represent 95% confidence intervals. Diamonds indicate global estimates of SDR. I-squared and P-values indicate heterogeneity among studies included in this meta-analysis. The studies are arranged by year of publication.

The pooled estimate for SDR was calculated to be 77%, with a 95% confidence interval of 69% to 85% (Figure 4). The overall heterogeneity among the studies was very high (I2 = 94.47%), which was also found to be statistically significant (p-value).

Discussion

Orthodontics as a specialty aims to address the problem of malocclusion after thorough diagnosis and treatment planning. To aid the orthodontist, cephalometry serves as an essential diagnostic aid [25]. However, accurate recognition of cephalometric landmarks is essential for proper diagnosis and treatment planning.

In a study conducted at Punjab University by Juenja et al. in 2021, it was observed that out of the several techniques employed during cephalometric landmark identification, either digital or manual, undoubtedly the digital methods, which were software-based such as AI, ML, CNN, etc., are not only energy efficient but also help to reduce the expenditure of time and are more efficient and accurate [28].

In this article, we report seven papers that used deep learning for automated landmark detection. Leonardi et al. in 2009 used AI-based Deep (CNN) to perform automated quantitative cephalometric measurements and identified cephalometric landmarks with a minimal difference; they were found within 0.59 mm, and the system performed well compared to the best literature benchmarks, with a reported accuracy of 76% [29]. In a similar study by Schwendicke et al. in 2021, deep learning showed consistent and high accuracy, but landmark prediction error centered around 2 mm, and the obtained proportion of landmarks within this threshold was 0.799 [30].

The above studies using automated cephalometric landmark identification represent a suitable alternative for diagnosis and detection of treatment planning progress where we need to superimpose frequently. Orthodontic treatment and progress require repeated cephalometric imaging and superimposition to access the progress of treatment. Therefore, automatic cephalometric landmark detection using AI is a more viable and alternative method because it predicts accuracy when repeated identification becomes essential.

In 2020, Hwang et al. concluded that AI-based identification of cephalometric landmarks seemed to be as accurate as the conventional method [5], whereas in 2021, Kim et al. proposed that the deep learning model could achieve better results than the manual method for certain landmarks [23].

During the 19th Biomedical Imaging Conference in 2022, they developed a new method of machine learning to detect landmarks automatically. They drew the conclusion that the conventional method not only drains energy but also consumes time. Apart from that, the risk of bias becomes manifold as there is variation not only between different observers but also between the observations done by the same observer at different times [31-33].

The above systematic review, which included seven relevant publications, has a couple of limitations. In order to identify cephalometric landmarks, the first method employed was automated using machine learning, and no comparisons with other semi-automatic landmark methods were made. Many studies were excluded because they were used to predict bony anomalies or because the full text was not available and valuable data could have been lost. Third, the included studies had many risks of bias. Lastly, it can also be stated that only those articles published in English were considered, and those that might have been published in other languages have not been considered.

## Conclusions

The outcome of this systematic review has led to the conclusion that AI, when employed for cephalometric landmark detection, has shown extremely positive and promising results as compared to manual tracing. This not only helps to improve the accuracy of diagnosis but also enhances clinical decision-making along with improved predictability of treatment prognosis. All of this helps the clinician render the utmost patient care by utilizing diagnostic aids with the minimum of errors. Thus, it can be stated that AI systems, as auxiliary tools, help save not only time but also energy. AI systems have evolved in a manner that will revolutionize dentistry as a whole and bring about phenomenal change, particularly in the orthodontic diagnosis and treatment planning phases. This is just the beginning of a journey that will provide us with numerous benefits in this era of machine learning and digitization.
